# Early synaptic dysfunction induced by α-synuclein in a rat model of Parkinson’s disease

**DOI:** 10.1038/s41598-017-06724-9

**Published:** 2017-07-25

**Authors:** Jenny-Ann Phan, Kathrine Stokholm, Justyna Zareba-Paslawska, Steen Jakobsen, Kim Vang, Albert Gjedde, Anne M. Landau, Marina Romero-Ramos

**Affiliations:** 1 Department of Biomedicine, NEURODIN AU IDEAS Center, Aarhus University, Wilhelm Meyers Allé 4, bldg. 1242, Aarhus C, 8000 Denmark; 2 Department of Nuclear Medicine and PET Centre, Aarhus University and Hospital, Norrebrogade 44, bldg. 10G, Aarhus C, 8000 Denmark; 3 Department of Neuroscience and Pharmacology, University of Copenhagen, Blegdamsvej 3, Copenhagen, 2200 Denmark; 4 Center of Neuroscience, University of Copenhagen, Copenhagen, Denmark; 5 Dept of Radiology & Radiological Science, Johns Hopkins University, Baltimore, MD USA; 6 Department of Neurology and Neurosurgery, McGill University, Montreal, Quebec Canada; 7 Neurosciences Research Center, Tabriz University of Medical Sciences, Tabriz, Iran; 8 Department of Clinical Medicine, University of Southern Denmark, Odense, Denmark; 9 Translational Neuropsychiatry Unit, Aarhus University, Skovagervej 2, Risskov, 8240 Denmark

## Abstract

Evidence suggests that synapses are affected first in Parkinson’s disease (PD). Here, we tested the claim that pathological accumulation of α-synuclein, and subsequent synaptic disruption, occur in absence of dopaminergic neuron loss in PD. We determined early synaptic changes in rats that overexpress human α-synuclein by local injection of viral-vectors in midbrain. We aimed to achieve α-synuclein levels sufficient to induce terminal pathology without significant loss of nigral neurons. We tested synaptic disruption *in vivo* by analyzing motor defects and binding of a positron emission tomography (PET) radioligand to the vesicular monoamine transporter 2, (VMAT2), [^11^C]dihydrotetrabenazine (DTBZ). Animals overexpressing α-synuclein had progressive motor impairment and, 12 weeks post-surgery, showed asymmetric *in vivo* striatal DTBZ binding. The PET images matched ligand binding in *post-mortem* tissue, and histological markers of dopaminergic integrity. Histology confirmed the absence of nigral cell death with concomitant significant loss of striatal terminals. Progressive aggregation of proteinase-K resistant and Ser129-phosphorylated α-synuclein was observed in dopaminergic terminals, in dystrophic swellings that resembled axonal spheroids and contained mitochondria and vesicular proteins. In conclusion, pathological α-synuclein in nigro-striatal axonal terminals leads to early axonal pathology, synaptic disruption, dysfunction of dopaminergic neurotransmission, motor impairment, and measurable change of VMAT2 in the absence of cell loss.

## Introduction

Parkinson’s disease (PD) is a neurodegenerative disorder characterized mainly by progressive loss of dopaminergic neurons from substantia nigra (SN). The pathological hallmark of PD is the presence of Lewy bodies and neurites predominantly composed by aggregated α-synuclein (ASYN) in the remaining neurons. Formation of neurotoxic oligomers of this protein is held to be the fundamental pathogenic event in both hereditary and sporadic forms of PD.

Genetic studies identified several point mutations of the ASYN gene (SNCA) carried by PD patients with autosomally dominant phenotypes^1^, including duplication and triplication of the SNCA locus described in families with autosomally dominant inheritance. Locus duplication is linked to sporadic PD with late onset, slow disease progression, and absence of prominent cognitive decline^2^, while SNCA triplication is linked to an aggressive phenotype, characterized by earlier onset, more extensive ASYN deposition, and more severe motor symptoms^3^. These findings imply that clinical features depend on the degree of SNCA gene expression. In support of this view, biochemical analyses reveal that SNCA triplication in PD subjects yields higher ASYN protein levels in blood and ASYN mRNA in brain, compared to controls, together with extensive ASYN deposition in the form of advanced molecular aggregates^4^.

Studies of genetic variability of the ASYN promoter region in sporadic PD identified regulatory elements in control of SNCA expression that explain the enhanced ASYN expression associated with susceptibility to sporadic onset in the absence of mutations or multiplication^5^. Together, the findings lead to the view that elevated expression of wild-type ASYN is sufficient to induce PD. Disruptions of synaptic transmission and dysfunctional dopamine release are held to be initial events that precede the neuronal cell death induced by ASYN. Indeed, models as transgenic animals with overexpression of ASYN show neuronal dysfunction in the absence of cell loss, including motor defects, electrophysiological changes in basal ganglia, and variability of dopamine release^6^.

Previously, we used local microinjections of recombinant adeno-associated viral vectors (rAAV) coding for human ASYN, to develop a model of early PD in rats. The condition of the rats mimicked pathological characteristics of PD more closely than rat models of the disease induced by toxins (e.g., 6-OHDA and MPTP), with gradual nigrostriatal degeneration, motor impairments, and presence of ASYN deposits in remaining neurons in rodent and primates^7, 8^. The motor defects of the model rats matched the degree of dopaminergic degeneration^7, 9, 10^, in proportion to the level of ASYN, determined by the type and titer of the viral vector^11^.

In the model, ASYN-induced changes of dopaminergic synaptic transmission are the primary events. Thus, the decline of evoked dopamine release in striatum is observed before any significant loss of dopaminergic striatal fibers^9, 10^, and early alterations of proteins involved in synaptic vesicle exocytosis precede the loss of dopaminergic cells^12^. The model is the result of a unique approach to the modulation of the neurodegenerative process responsible for the ASYN-induced neuropathology with and without loss of cells^13^.

Dihydrotetrabenzazine, DTBZ, is a ligand of vesicular monoamine transporter 2, VMAT2, and is considered a robust marker of dopaminergic integrity^14^. Recent studies using radiolabeled DTBZ revealed a reduced VMAT2 availability in striatum and SN in patients with PD^15^. DTBZ has been used to explore various experimental models of PD showing robust cell death both, in rodents and in monkeys, however *in vivo* DTBZ binding has not been investigated in ASYN models. Here, we test the hypothesis that elevated expression of wild-type human ASYN is sufficient to induce hemi-parkinsonism in rats *in vivo*, as evidenced by motor performance tests and positron emission tomography (PET) of [^11^C]DTBZ.

## Results

### Study design

**Figure 1:**
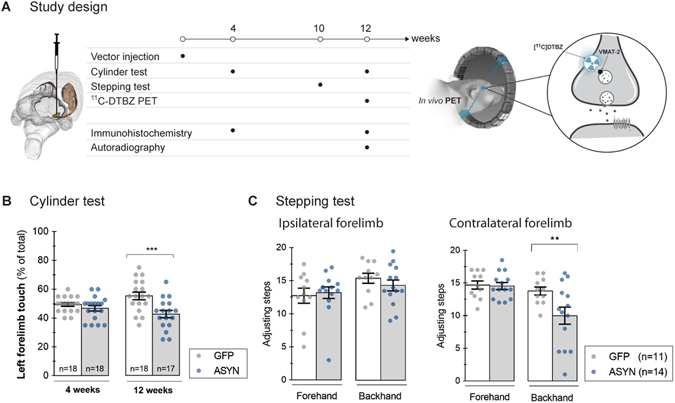
Study design And Motor Performance. The timeline of the study is illustrated in (**A**). Adult rats received a stereotactic unilateral injection of rAAV 2 µl (1·10^13^ gc/ml) in the right ventral midbrain. Motor performance was examined at 4 and 12 weeks after injection with the cylinder test, and the stepping test was done in a separate group at 10 weeks. A group of animals was killed at 4 weeks to evaluate transgene expression and to analyze the dopaminergic system by immunohistochemistry. Animals underwent *in vivo* [^11^C]DTBZ PET at 12 weeks and thereafter they were divided in two groups, killed and processed for *in vitro* autoradiography or for immunohistochemistry in order to correlate *in vivo* findings with anatomical data. Motor performance evaluated with the cylinder test (**B**) revealed impaired use of the ipsilateral forelimb us at 12 weeks in ASYN animals but not in GFP controls. Two way ANOVA (Interaction time & rAAV F(1, 67) = 5.3 P = 0.02; time effect F(1,67) = 0.14 P = 0.70; rAAV effect F(1,67) = 12.36 P = 0.0008) followed by Sidak’s correction for multiple comparisons. The stepping test was performed at 10 weeks (**C**), and no significant difference in the number of steps was found in the ipsilateral forelimb in any of the groups. However, ASYN overexpressing animals performed significantly less number of steps with the contralateral forelimb in the forehand direction (**C**). Two way ANOVA (Interaction direction & rAAV F(1, 46) = 4.18 P = 0.04; direction effect F(1,46) = 9.43 P = 0.003; rAAV effect F(1,46) = 4.88 P = 0.03) followed by Sidak’s correction for multiple comparisons **P < 0.01, ***<0.001. All data are presented as mean ± SEM.

We tested the hypothesis that synaptic changes occur before prominent cell loss in a rat model of hemi-parkinsonism in which we locally overexpressed human ASYN by unilateral injection of rAAV2/6-ASYN (under the synapsin-1 promoter with an enhanced element, see Methods for details). As control, rAAV2/6- green fluorescent protein (GFP) was injected in a separate group of animals. We tested the animals in drug-free motor tests, and by *in vivo* PET imaging of VMAT2 performed prior to the endpoint at 12 weeks. *Post-mortem* analyses included immunohistochemistry for the transgene and relevant dopaminergic markers, as well as autoradiography with the same ligand used for PET imaging (Fig. 1A).

### Unilateral motor defects with α-synuclein overexpression

We first evaluated the response of motor behavior to ASYN overexpression with two drug-free tests, the cylinder test at 4 and 12 weeks, and the stepping test at 10 weeks. No preferential use of the forelimbs was observed in any of the groups at 4 weeks (Fig. 1B). At 12 weeks, animals overexpressing ASYN had a significant preference for the use of the ipsilateral forelimb in the cylinder, compared to the GFP rats, indicating progressive unilateral basal ganglia impairment of the ASYN rats (Fig. 1B). The impairment was observed also at 10 weeks post-surgery, as measured with the stepping test. All animals took similar numbers of steps in the backhand direction with both forelimbs (Fig. 1C). However, compared to the GFP animals, ASYN expressing animals took significantly fewer steps with the contralateral forelimb in the forehand direction (Fig. 1C). This preference further suggested that ASYN overexpression induced the progressive motor impairment, while GFP overexpression did not.

### Asymmetric VMAT2 binding with prolonged α-synuclein overexpression

**Figure 2:**
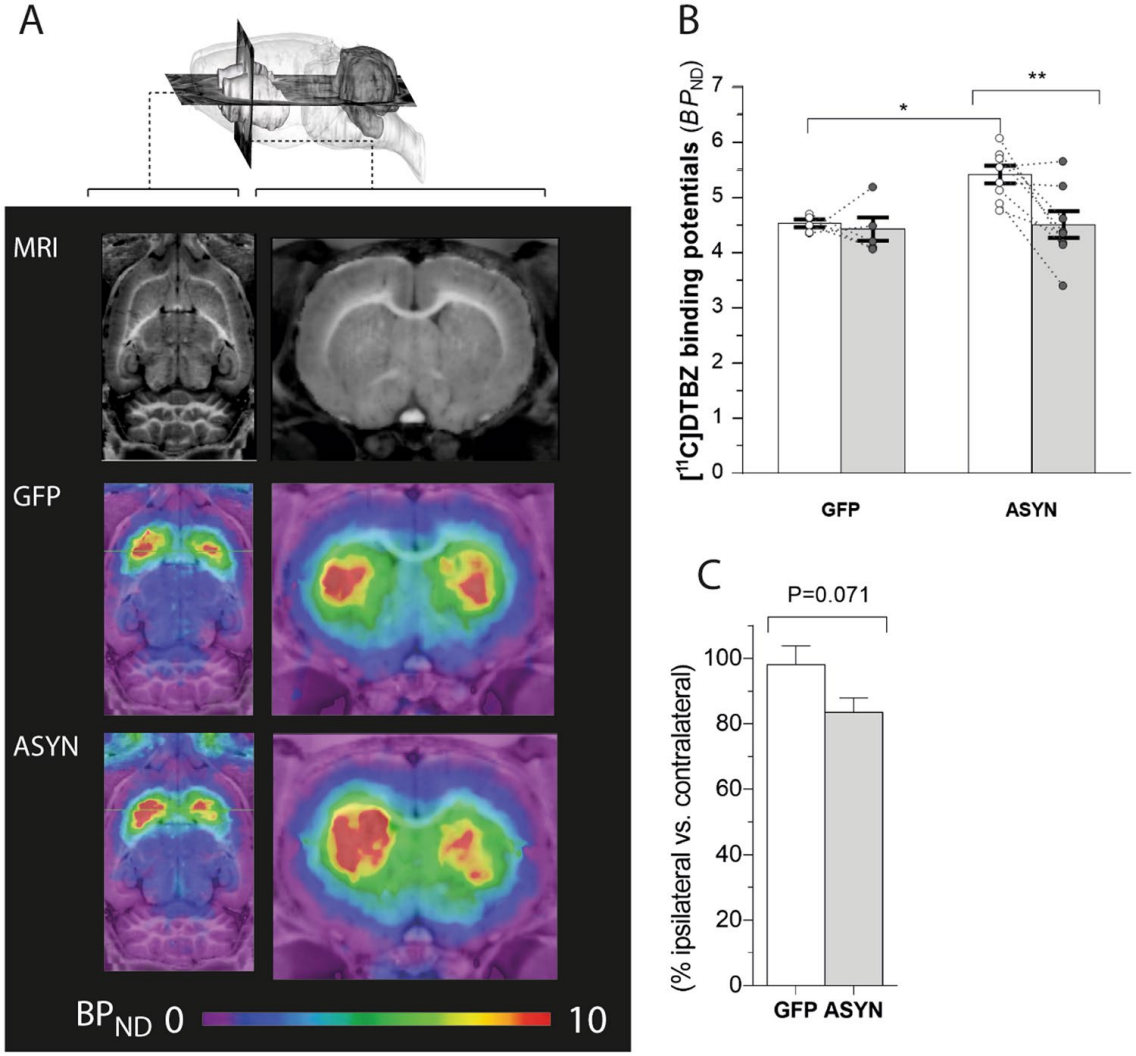
*In vivo* VMAT2 expression determined by [^11^C]DTBZ PET. Representative axial (**A**, left) and coronal (**A**, right) sections of MRI atlas (top row) and parametric maps of *in vivo* [^11^C]DTBZ binding potentials (*BP*_ND_) of GFP (middle row) and ASYN animals (bottom row) at 12 weeks post-surgery. While GFP animals showed similar binding in both sides of the striatum (middle row), a marked decrease in the binding in the ipsilateral side was observed in response to ASYN overexpression (bottom row). Quantification of [^11^C]DTBZ binding potentials (*BP*_ND_) in each side of striatum were done using Logan plot with cerebellum as reference input (**B**). DTBZ *BP*_ND_ was unchanged by GFP overexpression, whereas ASYN overexpression induced a significant reduction as compared to the contralateral side. In addition, we observed a significant difference of the DTBZ *BP*_ND_ in the contralateral side of the ASYN animals (n = 8) compared to the contralateral side in GFP rats (n = 5). Two-way ANOVA (Interaction: rAAV & side F(1,22) = 3.78 P = 0.06; rAAV effect F(1,22) = 5.49 P = 0.02; side effect F(1,22) = 5.93 P = 0.02); followed by Tukey’s multiple comparisons. *P < 0.05, **P < 0.01. (**C)** Shows the percent-wise difference of ipsilateral side relative to contralateral side in GFP controls and ASYN animals (unpaired t-test, t = 1.98, df = 11, P = 0.07).

To examine the viability of dopaminergic terminals *in vivo* in response to ASYN overexpression, we imaged the binding of tracer [^11^C]DTBZ in brain with PET. Animals expressing human ASYN in the right nigrostriatal neurons showed asymmetric binding of [^11^C]DTBZ in striatum 12 weeks post-rAAV injection (Fig. 2A and B). In contrast, GFP controls had symmetric DTBZ binding in striatum in the two hemispheres. Interestingly, the asymmetry induced by ASYN overexpression was associated with significantly higher [^11^C]DTBZ binding in the striatum contralateral to the side of SN injection, compared to the binding in the contralateral side in the GFP animals (Fig. 2A and B). Quantification of the binding potentials (*BP*_ND_) of [^11^C]DTBZ *in vivo* revealed significant asymmetry in response to ASYN overexpression, with an average of −16 ± 4.5% lower binding potentials in the ipsilateral sides compared to the contralateral side. In contrast, we noted no significant difference (−2 ± 5.8%) in response to GFP (Fig. 2C), suggestive of asymmetry of VMAT2 density is induced by ASYN overexpression in nigrostriatal fibers.

**Figure 3:**
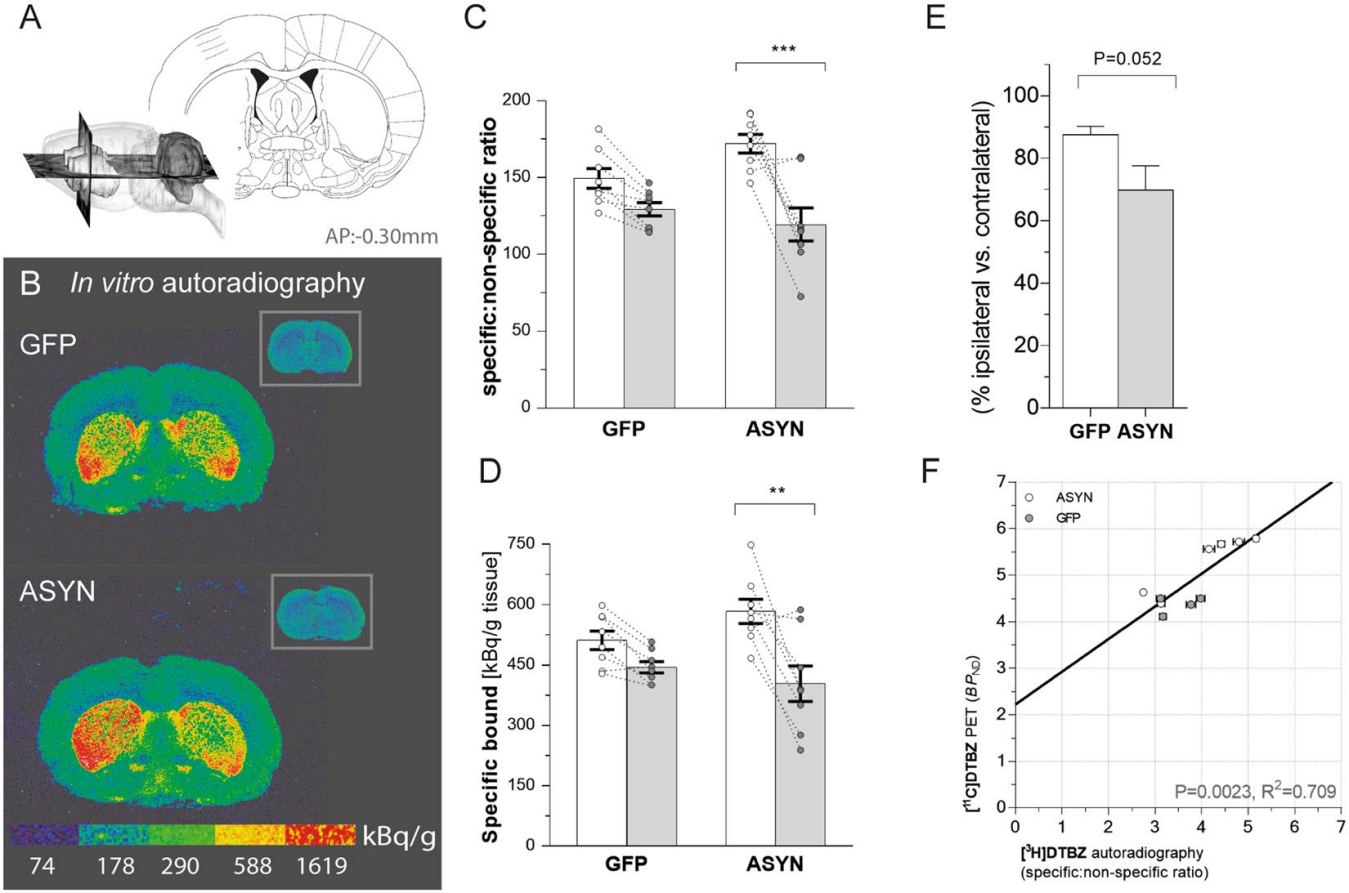
*in vitro* DTBZ[^3^H] autoradiography. Sketch showing the coronal section at −0.30 mm from bregma (from Paxinos & Watson, The rat brain in stereotaxic coordinates (Academic Press/Elsevier) used for autoradiography (**A**). Representative autoradiograms of [^3^H]-labeled DTBZ (**B**) and the insets show the non-specific binding in presence of cold DTBZ in striatal sections form GFP (top) and ASYN animals (bottom). Color scale indicates activity of bound radioligand in k Bq/g tissue. (**C**) Bars represent the specific:non-specific ratio of autoradiographic signal in each side of striatum per group. There was a significant reduction of the ligand binding in ASYN animals. Two-way ANOVA (Interaction: rAAV & side F(1,28) = 4.99 P = 0.03; rAAV effect F(1,28) = 0.76 P = 0.39; side effect F(1,28) = 24.85 P < 0.0001), followed by Tukey’s multiple comparisons (GFP n = 8 & ASYN n = 8). (**D**) Consistently, quantification of specifically bound [^3^H]DTBZ showed similar decrease in ASYN animals. Two-way ANOVA (Interaction: rAAV & side F(1,28) = 3.52 P = 0.07; rAAV effect F(1,28) = 0.27 P = 0.6; side effect F(1,28) = 17.08 P = 0.0003); followed by Tukey’s multiple comparisons. *P < 0.05, **P < 0.01, ***P < 0.001. (**E**) Shows the data as percentage of the contralateral side (unpaired t-test, t = 2.13, DF = 14, P = 0.052). The correlation of *in vitro* autoradiography and *in vivo* PET DTBZ binding (**F**) yielded a significant linear relationship (R^2^ = 0.71, P < 0.005). In F: each point corresponds to individual estimates from animals, which underwent both methods. Horizontal standard error bars represent the variation of triplicate values for each individual rat in the autoradiography quantification; and the vertical error bars, the individual mean square error in the linear regression for binding potential calculation.

The comparative *in vitro* autoradiography of striatal brain sections with tritium-labeled DTBZ had greater spatial resolution than PET, and autoradiograms of [^3^H]DTBZ binding revealed significantly asymmetric binding in ASYN animals but not in GFP animals (Fig. 3B). Quantification of the specific binding (Bq/g tissue) (Fig. 3C) and the specific-to-non-specific ratios (Fig. 3D) revealed decline of *in vitro* DTBZ binding by 30±7.6% in response to the ASYN overexpression, compared to the intact hemisphere. As with the *in vivo* results, *in vitro* autoradiography revealed no significant decline in the GFP group (−2 ± 2.3%) (Fig. 3E). In contrast to PET of [^11^C]DTBZ, we found no significant difference of the [^3^H]DTBZ binding between the contralateral sides of ASYN vs. GFP animals (Fig. 3C and D).

To determine the correspondence of DTBZ binding measures *in vivo* and *in vitro*, we compared the [^11^C]DTBZ *BP*_ND_ estimates obtained with PET (defined as the ratio of specifically-to-non-specifically bound radiotracer at steady-state) with the specific-to-nonspecific binding ratios of [^3^H]DTBZ obtained with autoradiography. The linear correlation of the *in vivo* and *in vitro* measures from brains with both procedures was highly significant (R^2^ = 0.709, P = 0.0023; Fig. 3F). The correlation yielded an even better linear fit when the percentage *in vivo* and *in vitro* differences of ipsilateral and contralateral hemisphere values were compared (R^2^ = 0.79, P < 0.05, not shown).

### Progressive accumulation of pathological α-synuclein in dopaminergic fibers

**Figure 4:**
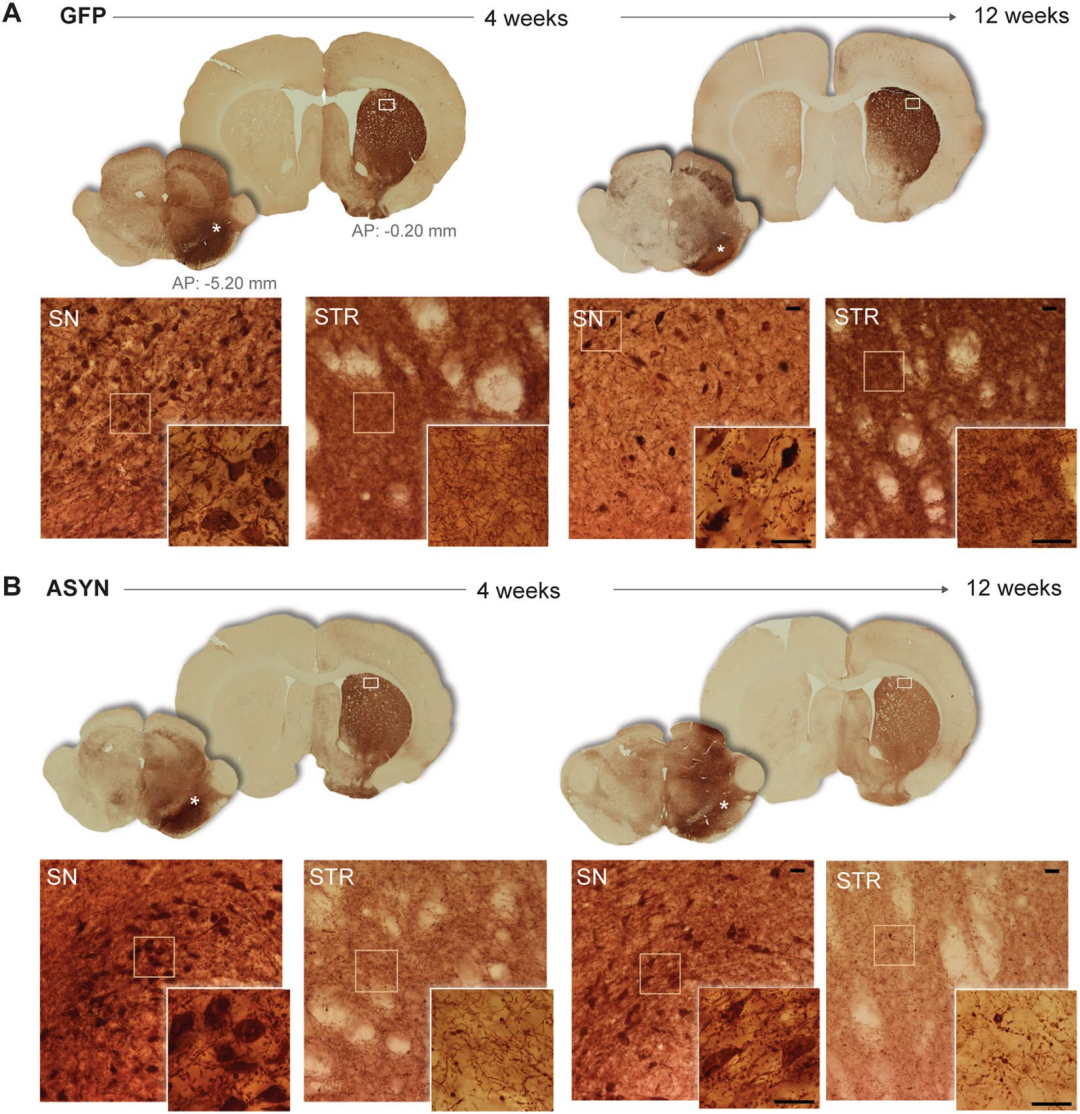
Unilateral transgene expression. (**A**) Schematic views of the coronal sections of substantia nigra (SN) and striatum used for evaluation of transgene expression by immunohistochemistry against GFP (**B**) or human ASYN (**C**). (**A**). Photomicrographs of the areas marked with asterisk in the SN sections or a frame in striatal sections are shown in the bottom rows (**B** and **C**); inserts display higher magnifications of details framed in the photos. Robust transgene expression was found in the nigrostriatal system at 4 and 12 weeks in both GFP (**B**) and ASYN animals (**C**). In SN, numerous cell bodies resembling neurons expressed the transgene, and this persisted in time in both groups. In striatum, GFP control animals displayed dense immunostaining of the transgene in the striatal terminals, which appeared healthy and of slightly higher intensity as time progressed (**B**). In ASYN animals as early as 4 weeks we observed pathological swelling and thickening of fibers in striatum (**C**). Scale bars in the inserts from 12 weeks photos correspond to 25 um, which also applies to the images from 4 weeks.

Injection of rAAV resulted in robust and persistent expression of the transgene in the nigrostriatal pathway, as revealed by immunohistochemistry. The injected ipsilateral midbrain displayed high transgene expression at 4 and 12 weeks (Fig. 4A–C). The transgene was expressed efficiently in nigral cell bodies that resembled neurons, with no indication of staining observed in glia-like cells, or in the contralateral side, as predicted. Positive immunoreactivity in the ipsilateral striatum implies that ASYN and GFP both were synthesized by transfected dopaminergic neurons and delivered by anterograde transport to the synaptic terminals. The striatal GFP staining was homogenous, with no indication of pathology or change of fiber density with time (Fig. 4B). In contrast, striatal fibers in animals overexpressing ASYN appeared pathological with dystrophic terminal swellings filled with ASYN (Fig. 4C). The density of ASYN^+^ fibers decreased with time, in parallel with the loss of terminals, while the dystrophic swellings grew.

**Figure 5:**
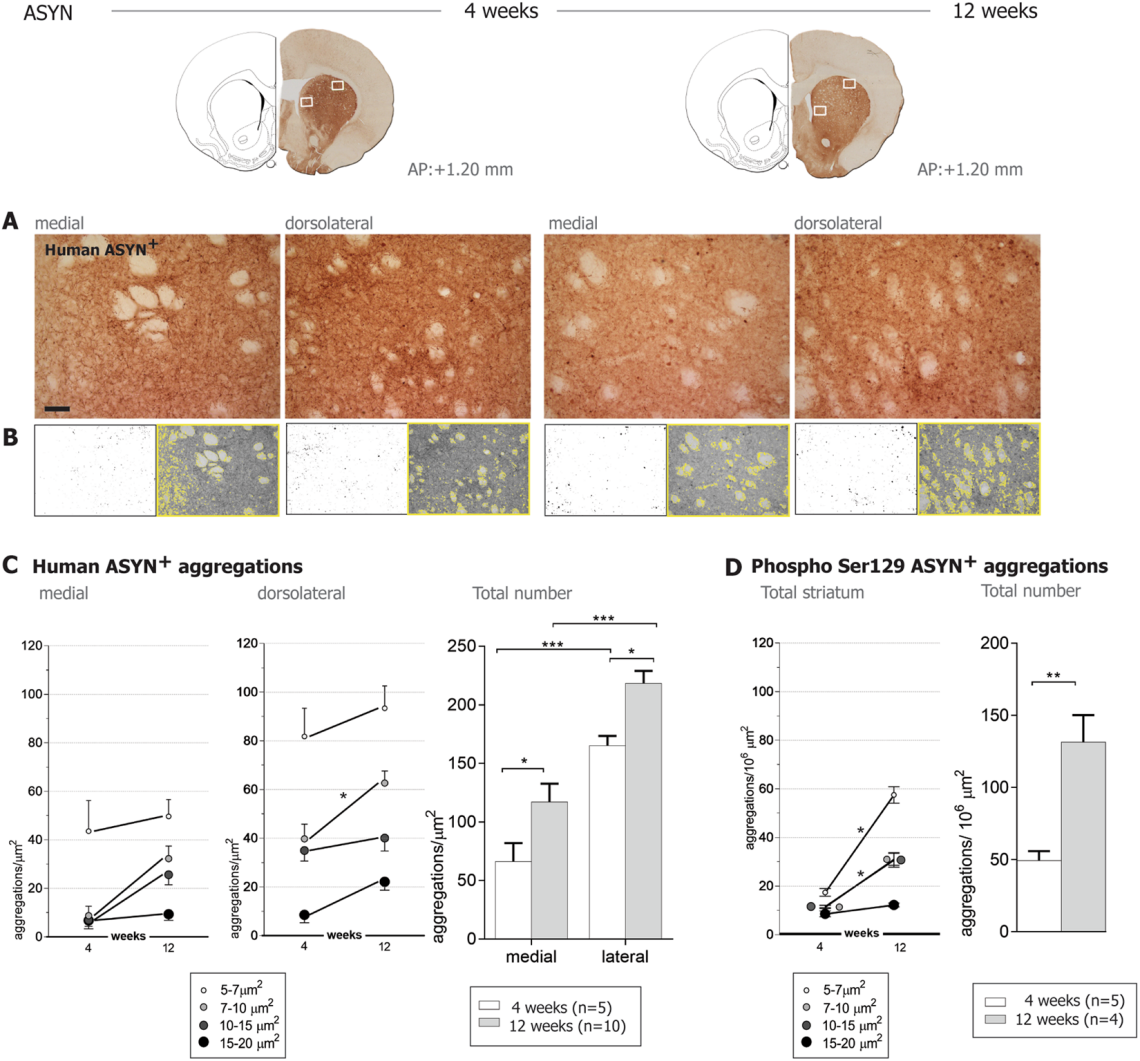
Quantification of ASYN^+^ aggregates in striatum. Upper row shows an example of the striatal sections immunostained against human ASYN and the representative areas at the medial and dorsolateral striatum that were photographed (shown in **A**) and used for the quantification of ASYN^+^ inclusions in striatal fibers. (**B**) Illustrates the quantification method: left panels show masks of inclusions > 5 µm^2^ generated by a grey scale threshold in the software ImageJ. The area covered by ASYN^+^ immune-reactivity was segmented from the myelin-rich areas (showed by the yellow outline in the right images) and used for normalization. (**C**) Show numbers of ASYN^+^ swellings per µm^2^ by size and total numbers per area and time (4 weeks n = 5, 12 weeks n = 10). Dorsolateral striatum exhibited more ASYN^+^ aggregates than the medial striatum; but the number of aggregates increased with time in both areas. Two-Way ANOVA (Total number, time effect F(1,26) = 12.41 P = 0.001; area effect F(1,26) = 45.94 p < 0.0001) followed by Sidak’s multiple comparisons. (**D**) Analysis of Ser129 phosphorylated ASYN (ASYN S129)^+^ inclusions in the total striatal area revealed a significant increase of small and medium-sized ASYN S129^+^ with time, but not of the big ones (15–20 µm^2^). Two-Way ANOVA (Dorsolateral, Interaction Time&size F(3,28) = 7.88 P = 0.006, time effect F(1,28) = 60.04 P < 0.0001; size effect F(3,28) = 17.54 p < 0.0001) followed by Sidak’s multiple comparisons. In the right graph, total number of ASYN S129^+^ aggregates increased with time (Unpaired t-test F (3,4) = 6.534, P = 0.002). Notice fewer total ASYN S129^+^ aggregates were found per µm^2^ compared to ASYN^+^ aggregates (see scale difference in y axis in **C** and **D**), thus only a small portion of ASYN^+^ accumulations contained S129 phosphorylated ASYN. *P < 0.05, **P < 0.01. Scale bar = 60 µm applies to all. Brain sections drawings are modified from Paxinos & Watson, The rat brain in stereotaxic coordinates (with permission Academic Press/Elsevier).

In order to confirm the progressive accumulation of the ASYN^+^ inclusions in striatal terminals, we quantified them at 4 and 12 weeks, noting that both size and number of the pathological swellings/inclusions rose with time (Fig. 5A–C). The number of ASYN inclusions was higher in the dorsolateral than the medial striatum, consistent with a regional difference.

### Pathology and loss of striatal terminals without nigral neuron loss

**Figure 6:**
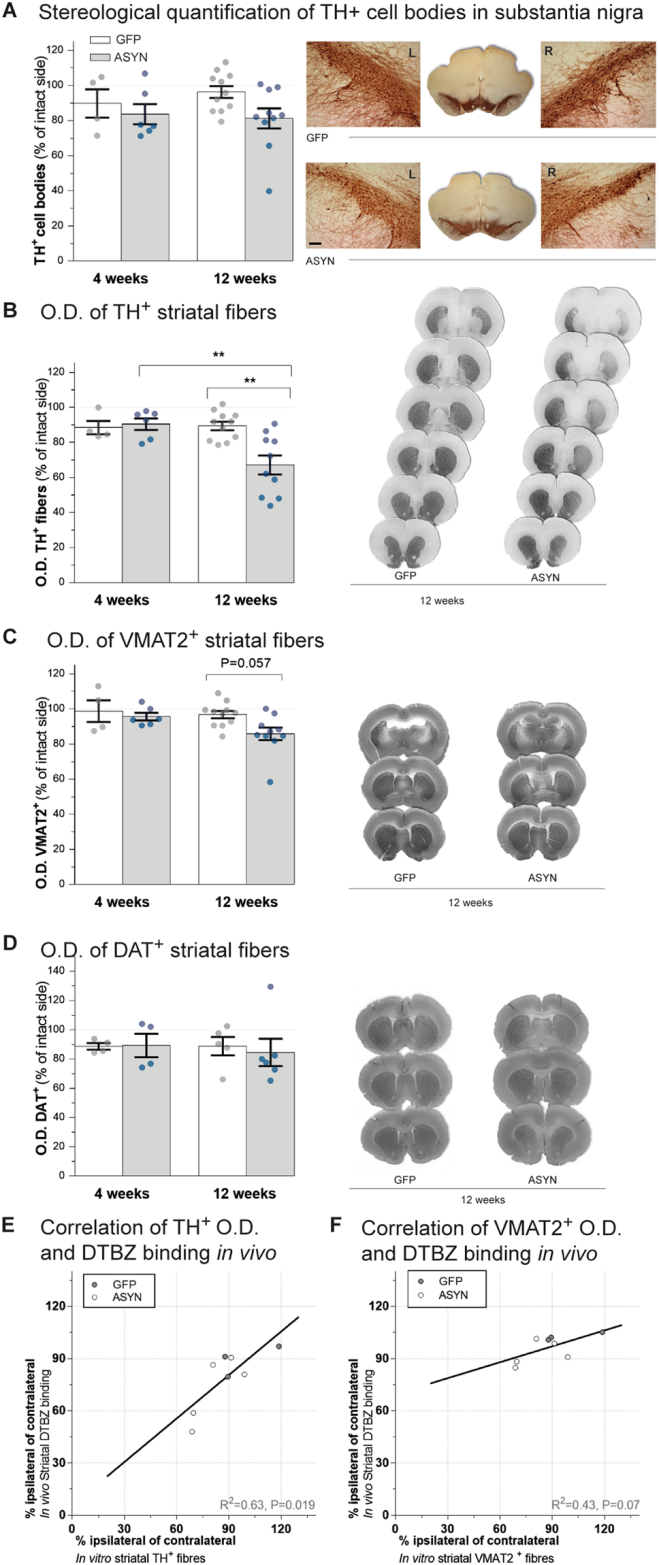
TH and VMAT2 histology. Stereological quantification of TH^+^ cell bodies in SN in animals overexpressing GFP or ASYN revealed no significant cell loss in any groups (**A**) and no differences were found between both sides of SN in GFP (top right in **A**) or ASYN animals (bottom right in **A**) (Two way ANOVA, rAAV effect F(1,27) = 3.32 P = 0.079). Striatal dopaminergic innervation was also assessed (**B**–**D**). Bar graphs show semi-quantitative optical density (O.D.) analysis of TH^+^ (**B**), VMAT2^+^ (**C**) or DAT (**D**) immunostaining in striatum per group and time. A significant reduction of TH^+^ terminals was found in response to ASYN overexpression at 12 weeks (left **B**). Representative TH immunostained sections from ASYN animals show a more severe loss of TH immunoreactivity in dorsal and caudal areas of striatum than ventral areas (right **B**) Two way ANOVA (Interaction time & rAAV F(1, 27) = 6.83 P = 0.01; time effect F(1,27) = 5.82 P = 0.02; rAAV effect F(1,27) = 4.75 P = 0.03), followed by Tukey’s multiple comparisons. VMAT2 staining of striatal sections (**C**) revealed a smaller reduction of VMAT2^+^ terminals in ASYN animals at 12 weeks (P = 0.057). Two way ANOVA (Interaction time & rAAV F(1, 27) = 1.23 P = 0.27; time effect F(1,27) = 2.80 P = 0.10; rAAV effect F(1,27) = 3.94 P = 0.057). (**D**) DAT immunostaining did not show any differences. Remarkably, the densitometry analysis of TH immunoreactivity in striatal terminals was significantly correlated with *in vivo* DTBZ binding (**E**), whereas the VMAT2 immunoreactivity did not significantly correlate with *in vivo* DTBZ binding (**F**). (GFP 4w, n = 4; 12w, n = 11 & ASYN 4w, n = 6; 12 w, n = 10); *P < 0.05, **P < 0.01. Scale in A 120 µm applies to all.

Stereological quantification of surviving dopaminergic tyrosine hydroxylase (TH)^+^ cell bodies in SN (Fig. 6A) revealed minimal cell loss in response to ASYN (−16.3% and −18.7% at 4 and 12 weeks respectively) that was not significantly different from the loss in GFP controls (−10.2%, and −3.6%).

Previous studies suggested that dopaminergic degeneration begins in the dopaminergic terminals. With this model, we previously showed that it is possible to achieve ASYN expression in the nigrostriatal pathway associated with significant loss of dopaminergic terminals in striatum without loss of nigral neurons^8, 13^. The densitometry of immunostained striatal sections for TH and VMAT2 revealed progressive loss of TH^+^ projections in striatum in the animals that overexpressed ASYN (Fig. 6B). At 4 weeks, we found no significant change of the striatal TH^+^ density in the ASYN rats and they appeared similar to GFP controls. However, at 12 weeks post-surgery, the ASYN animals presented significant loss of 32.8% of TH^+^ striatal fibers in the ipsilateral side (**P < 0.01). In contrast, GFP animals retained similar levels of TH^+^ striatal fiber density to levels found at 4 weeks (−10.6%) (Fig. 6B). The loss of terminals in response to ASYN overexpression was more prominent in caudal and dorsal areas of striatum. In contrast, the GFP group had homogenous TH expression in both hemispheres throughout the extent of the striatum (Fig. 6B). Remarkably, the TH^+^ immunoreactivity in striatal projections correlated significantly with *in vivo* DTBZ binding (Fig. 6D).

The densitometry of striatal VMAT2^+^ immunostaining had a pattern similar to that of the TH^+^ staining, with smaller reduction of VMAT2^+^ fiber density that did not reach statistical significance (P = 0.057)(−14.1% for ASYN vs. −3.2% for GFP) (Fig. 6C). Densitometry analysis of the dopamine transporter (DAT)^+^ immunostaining in frontal striatum did not reveal any significant change between the groups (Fig. 6D, please note that due to lack of samples, we did not include in the DAT analysis in the caudal striatal section).We noticed that the animals with higher cell TH^+^ loss also had clearly decreased VMAT2^+^ striatal fiber density; however, animals with minimal cell TH^+^ death had no major decrease of striatal VMAT2 density, despite the substantial decrease in TH^+^ fiber density. This suggests that ASYN expression induced a greater decrease of TH expression than of VMAT2 (or DAT) proteins, suggesting that the TH^+^ density decrease may be the result not only of the loss of dopaminergic terminals, but also of possible down-regulation of the protein or axonal terminal dysfunction in response to the ASYN abundance.

**Figure 7:**
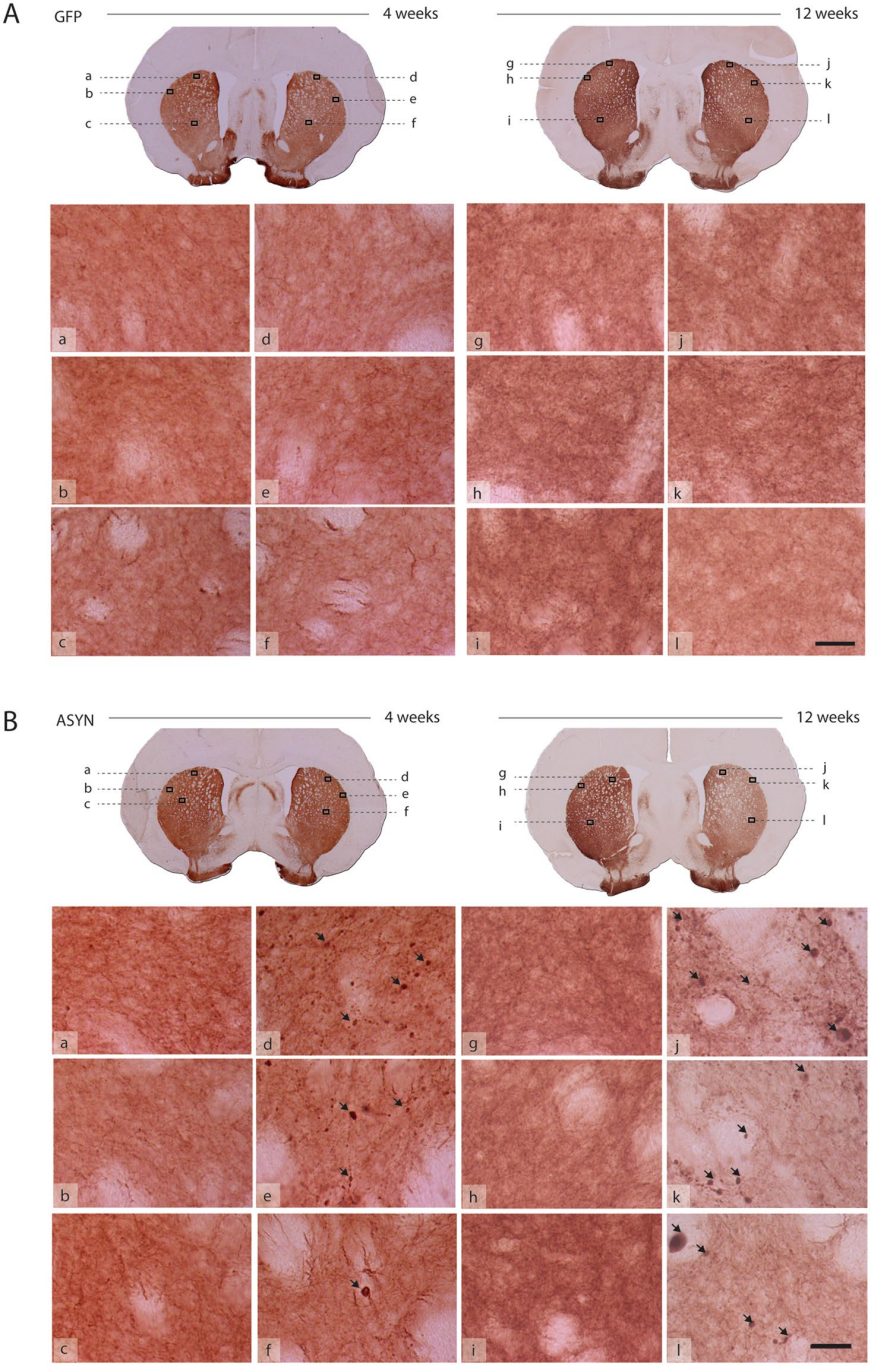
Pathology in striatal fibers. TH immunostained striatal sections from GFP (**A**) and ASYN animals (**B**) processed for histology at 4 weeks (left; a–f) or 12 weeks (right; g–l) post-surgery. Each section showed in low magnification in the upper row. Several high-power images are presented to reveal details of the fibers. GFP controls display healthy fibers of homogenous morphology in both striata at all time points (**A**). Notably, ASYN animals exhibit pathological TH^+^ formations (arrows) in the injected side at 4 weeks (**B**; d–f) and more severe pathology was observed later at 12 weeks (**B**; j–l; arrows). In comparison, the contralateral non-injected side displays homogenous distribution with no pathology (**B**; a–c and **B**; g–i), which was similar to the healthy morphology found in GFP controls. Scale bar = 30 µm applies to all high magnification photos.

We evaluated TH^+^ striatal fibers microscopically and noted that ASYN animals had pathological TH^+^ terminal swelling as early as at 4 weeks but more prominently and abundantly at 12 weeks (Fig. 7B). In size and number, the abnormal TH^+^ swellings in ASYN animals resembled the striatal ASYN^+^ inclusions described above (Fig. 4B). GFP overexpressing terminals appeared similar to contralateral terminals at all times with TH immunostaining (Fig. 7A) and other markers analyzed (not shown). VMAT2 immunostaining did not reveal the numerous pathological terminals seen with antibodies against TH or human ASYN (Supplementary Fig. 1A). Occasional rare VMAT2^+^ swellings were observed in striatum of animals with overt pathology as numerous swellings containing ASYN and TH. Furthermore, these axonal swellings were not visible upon DAT immunostaining (Supplementary Fig. 1A). The injected side was indistinguishable from the control side (not shown). The data suggests that ASYN overexpression leads to its abnormal accumulation in terminals, and it quickly leads to recruitment or pathological deposition of TH, but not initially of VMAT2^+^ structures only recruited in some of these swellings. This intracellular event does not induce, at least initially, any type of pathological structures containing DAT, probably due to the localization of this protein in the cytoplasmic membrane vs. the cytoplasmic localization of the other two. Confocal analysis of the striatal terminals confirmed that the expression and accumulation of ASYN occurred in the dopaminergic TH^+^ terminals in striatum (Supplementary Fig. 1B). The ASYN^+^ pathological swellings co-expressed TH and ASYN in most cases regardless of the size of the inclusion. However, only when ASYN^+^ inclusions were large did they have overt VMAT2^+^ staining (Supplementary Fig. 1B).

### Insoluble and phosphorylated α-synuclein both accumulate in dopaminergic terminals

**Figure 8:**
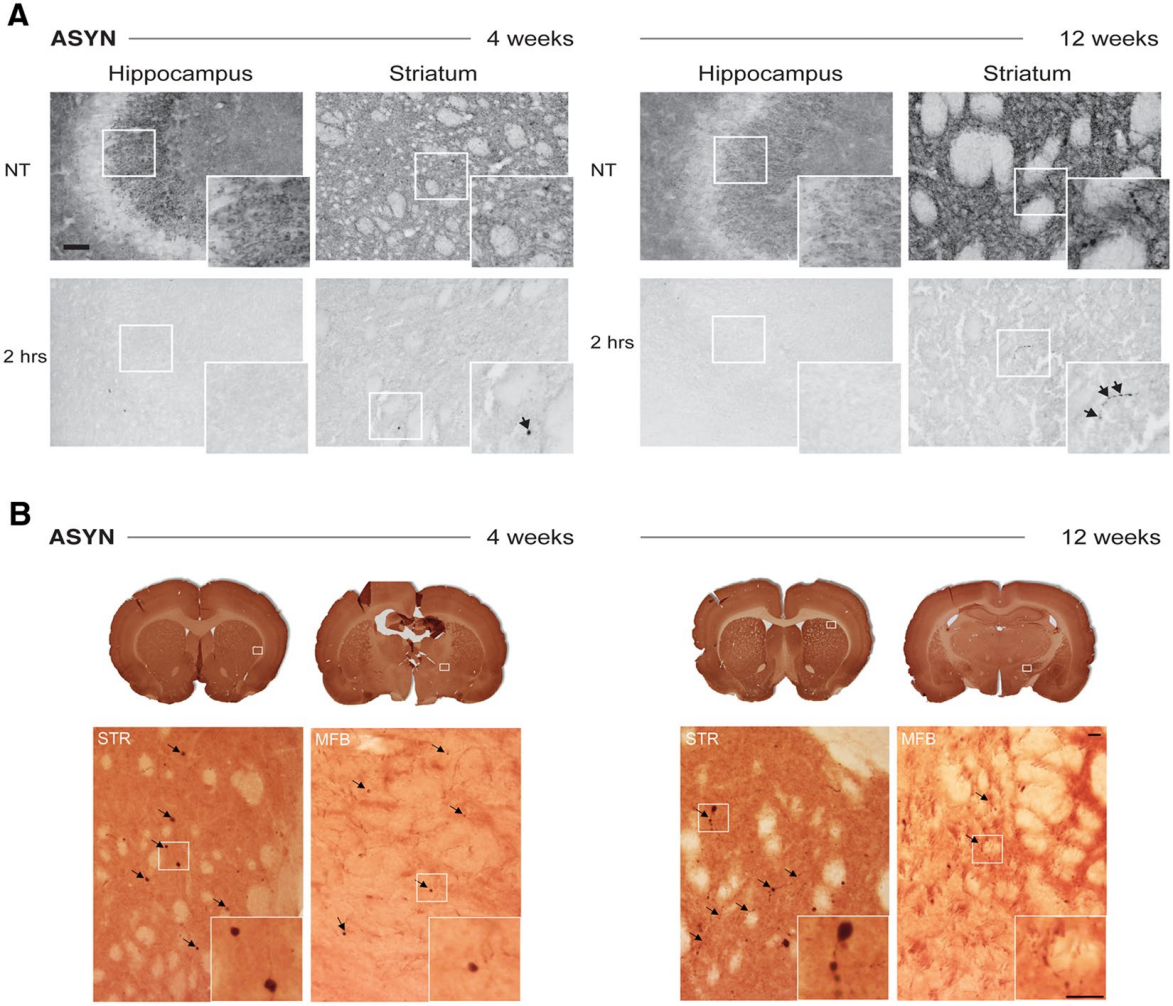
Pathological α-synuclein is present in axonal swellings. (**A**) The effect of proteinase K treatment on the hippocampus and striatum from ASYN animals at 4 and 12 weeks post-surgery. Sections were incubated with KPBS (NT; top row) or Proteinase-K in KBPS for 2 hours (2 hrs; bottom row), and then immunostained with an antibody recognizing both human and rat ASYN; inserts display higher magnifications of details framed in the photos. The hippocampus showed abundant endogenous ASYN^+^ signal in the CA3 that disappeared after 2 hours of proteinase K treatment. However, proteinase-K-resistant ASYN^+^ aggregates (arrows) were found in striatum from animals at 4 and 12 weeks. At 4 weeks, the proteinase-K resistant formations appeared as round scattered formations in ASYN animals (arrow in inset). After 12 weeks, we observed thick fibers and rosary-like ASYN^+^ formations, which were resistant to proteinase K digestion (arrows in inset). Scale bar = 60 µm applies to all. (**B**) P-Ser129-ASYN immunostained frontal and caudal striatal sections from ASYN animals 4 weeks (left), and 12 weeks (right) post-surgery. For each section showed in low power (top row) a high power image is presented in the bottom row to reveal details of the immunostaining in the striatum and medial forebrain bundle (MFB). In addition, each inset shows details of the P-Ser129-ASYN^+^ structures shown in the high power photos. While at 4 weeks, single round P-Ser129-ASYN^+^ inclusions were found in thin fibers (arrows and inset) in striatum, at 12 weeks we could find several of those round inclusions per fiber, which appeared as a rosary-like structures (arrows and inset). We also observed immunostaining in the MFB, with some single round inclusions at 4 weeks, that were smaller but more numerous at 12 weeks. Scale bar = 20 µm.

To determine the insoluble nature of the accumulated ASYN, we subjected selected representative striatal brain sections to digestion with proteinase K. As reference area for endogenous ASYN, we chose sections containing CA2-CA3 areas of hippocampus that had high expressions of the endogenous protein^16^. On-slide mounted sections were subjected to two hours of proteinase-K digestion, enough time to achieve total disappearance of endogenous ASYN (Fig. 8A). However, at this time, striatal sections of ASYN- overexpressing animals still contained ASYN^+^ proteinase-K-resistant inclusions in the ipsilateral hemisphere, confirming the insoluble character of the contained ASYN (Fig. 8A). These inclusions appeared as round ASYN^+^ isolated structures at 4 weeks, while they presented with rosary-like morphology at 12 weeks (i.e. several swellings in the same fiber), suggesting a progression of the pathological aggregation in individual terminals (Fig. 8A).

In parallel, we immunostained sections for phosphorylated -ASYN at serine129 residue (P-Ser129 ASYN^+^), which is highly accumulated in PD brains and it is considered a marker for pathological ASYN accumulation^17^. We observed scattered immunostained round structures at 4 weeks and some thin fibers in the ipsilateral striatum of rats overexpressing ASYN (Fig. 8B). At 12 weeks the fibers containing P-Ser129-ASYN became thicker and we observed not only single round structures, but also multiple inclusions per fiber resembling rosary-like formations as mentioned above (Fig. 8B). Quantification of the round P-Ser129-ASYN^+^ structures revealed an increase of the total number of aggregates with time (Fig. 5D), which was associated with increase in the small and medium (5–15 µm^2^) size aggregates, rather than the increase in those of larger size (15–20 µm^2^), according to the microscopy analysis. We observed fewer total number of P-Ser129-ASYN + than ASYN + aggregates (Fig. 5C and D), suggesting that ASYN is first pathologically accumulated and later phosphorylated, or that the aggregates later recruit P-Ser129-ASYN as previously suggested^18^. We also analyzed P-Ser129-ASYN immunostaining in the axonal track of the medial forebrain bundle (MFB), and we found neuropil-like structures and round formations containing the phosphorylated ASYN, suggesting that the pathological phosphorylation was also present in axons through the MFB and not only at the terminal (Fig. 8B).

Since these structures resembled axonal spheroids, we also co-immunostained for proteins known to accumulate in these types of pathological formations: neurofilaments, amyloid precursor protein (APP) and as a representative mitochondria protein, HSP70^19^. Our analysis did not reveal any overt accumulation of APP or neurofilaments in the ASYN + aggregates (Supplementary Fig. 2). Occasionally, we observed positive co-localization of HSP70 in the larger ASYN^+^ inclusions (Supplementary Fig. 2C). We should note however, that when we analyzed the axonal track of the MFB, we found single ASYN^+^ structures per field that also expressed neurofilament (Supplementary Figure 3).

## Discussion

We show here that elevated expression of human ASYN in the absence of neuronal loss is sufficient to induce hemi-parkinsonism in animals and that the changes are detected *in vivo* by non-invasive PET. We identified early synaptic changes that occurred before prominent cell loss in the model of hemi-parkinsonism in rats in which human ASYN (or GFP in the control group) was expressed locally in the nigrostriatal pathway. We examined animals by drug-free motor tests and *in vivo* PET imaging of VMAT2 prior to the endpoint at 12 weeks. *Post-mortem* analyses included immunohisto-chemistry for transgenes and relevant dopaminergic markers, tests of the ASYN status and pathology, as well as autoradiography with the same ligand used for PET imaging. We used adult transgenesis by intranigral injections of rAAV to express ASYN in midbrain at levels sufficient to induce abnormal accumulation of the human ASYN (as compared to the described synaptic endogenous ASYN) in dopaminergic neurons without overt cell loss. With this design, we found that progressive pathological accumulation of insoluble and Ser129-phosphorylated ASYN leads to dystrophy of dopaminergic fibers and to terminal loss that results in motor defects, revealed by the stepping test as early as after 10 weeks, and by the cylinder test at 12 weeks after ASYN overexpression. *In vivo* PET revealed significantly asymmetric binding of the ligand to VMAT2 at 12 weeks, suggesting synaptic disruption triggered by ASYN abundance and pathological aggregation. In support of the *in vivo* PET data, *in vitro* autoradiography and immunostaining of VMAT2 and TH confirmed the results.

VMAT2 mediates the uptake of cytosolic dopamine into vesicles and therefore is less subject to interference from reuptake inhibitors than other monoamine transporters, such as the DAT^14, 20^. Therefore, we hold VMAT2 imaging to be a better marker of the status of the dopaminergic neurotransmission than DAT imaging. Notably, DTBZ binding is decreased in PD patients^21, 22^ and after dopaminergic cell loss in animals induced by 6-OHDA^23–25^, MPTP^26^, lactacystin^27^ or by injections in SN and striatum of Lewy body extracts from PD brains^28^. But this is the first report of DTBZ imaging with PET of animals with ASYN overexpression in neurons. A recent paper reported a decrease in DAT using PET imaging that coincided in time with the dopaminergic cell loss induced by overexpression of A53T-ASYN using rAAV^29^. Here, however, we demonstrate that PET images of DTBZ binding reveal changes in dopaminergic neurotransmission induced by degenerating neurons that overexpress ASYN in the absence of neuronal loss. This property makes DTBZ a robust early marker of the integrity of the nigrostriatal system, which is applicable across different animal models (genetic and toxin-based) and different levels of dopaminergic neurodegeneration (early and advanced stages), in agreement with the recent studies of PD patients that suggested the use of DTBZ as an efficient marker of disease severity^15^.

The decline of DTBZ binding in the ipsilateral vs. the contralateral side in the ASYN animals was more pronounced *in vitro* than *in vivo*. The underestimation *in vivo* is attributable to partial volume effects of the rat brain, in combination with the limited spatial resolution of positron emission tomograms. The *in vivo* PET images and *in vitro* autoradiographs of the same ligand had significant linear correlation that agrees with a previous report of DTBZ binding in 6-OHDA-lesioned rats with a correlation of r^2^ = 0.64 and P < 0.005^25^, comparable to the results of the present study in which we correlated autoradiography (kBq/g tissue) with PET. When we correlated the specific-to-non-specific ratios obtained by autoradiography with the PET of DTBZ binding potentials, the correlation yielded a better fit. The improved fit suggests that the specific to non-specific ratio is more suitable for comparison with the *in vivo* binding potentials, defined as the ratio of specifically bound to non-displaceably bound radioligand at steady-state^30, 31^.

The *post-mortem* analysis revealed signs of pathological damage in striatal terminals that occurred with a trend towards a decrease in the VMAT2^+^ striatal fibers and a significantly higher loss of TH^+^ fibers in striatum, despite the absence of nigral cell loss. Remarkably *in vivo* DTBZ PET only correlated significantly with TH but not with VMAT2 immunoreactivity. This suggests PET and autoradiography may be more robust techniques to detect functional changes that can otherwise be missed using immunohistological techniques. Therefore, histology may be more suitable to explore protein/structural disruptions. The correlation between DTBZ PET-and TH immunostaining could suggest that TH changes are involved in both functional and structural disruption in the ASYN model. It has been shown that ASYN overexpression *in vitro* decreases TH mRNA and protein levels^32–34^. In addition, ASYN might act as a regulator of TH expression by nuclear translocation and binding to the promoter region^35^. We propose that the total loss of striatal TH immunoreactivity in the absence of SN neuronal death is caused by loss of terminals but also down-regulation of the enzyme, since decline of VMAT2 immunoreactivity was not commensurate. At this early stage, our data suggest no significant changes in DAT. Furthermore, we observed that pathological ASYN^+^ aggregations in terminals developed with time, in number and size. In agreement with our previous work^36^, we noted that there were fewer VMAT2^+^ pathological accumulations than TH^+^ or human ASYN^+^ accumulations, suggesting that abnormal assembly of VMAT2 is secondary to that initiated by ASYN, followed by TH deposition and lastly, but not always, by VMAT2 to finally result in axonal loss. As previously reported in the model, we did not observe any accumulation of DAT in these swellings, maybe due to localization of DAT in the cytoplasmic membrane vs. the intracytoplasmic location of TH and VMAT2^9^.

Since our samples were used for histology, we could not analyze levels of expression of human ASYN vs. the endogenous ASYN, thus we cannot conclude how comparable our model is with respect to the 2-fold increase found in PD patients with gene multiplication^4^. However, our findings show that human ASYN overexpression initially leads to synaptic disruption that results in motor defects, metabolic changes (i.e., loss of TH expression) and degeneration of terminals, even in the absence of significant neuron loss. This course of events is consistent with the common hypothesis of onset of neurodegeneration in terminals. We as well as others previously reported that ASYN overexpression induces loss of cells in SN^8, 13, 37–39^ that depends on the type (mutated or WT) and level of ASYN expression, as suggested by transgenic ASYN mouse models^40^. However, ASYN levels can be reached that result in terminal pathology, disrupted neuronal transmission, and behavioral defects, while the dopaminergic neurons survive in SN^8, 13, 39^, as also observed in other neuronal populations such as the dopaminergic neurons in VTA^41^. Therefore, terminal loss does not always correlate with an equivalent neuronal cell body loss. Indeed, in humans shortly after PD diagnosis, putamen can be almost completely devoid of TH axons, while numerous neuronal cell bodies are still found in SN pars compacta (SNc)^42^. This suggests that the neurodegenerative process in PD starts in terminals with axons degenerating first, which is confirmed by different signs of axonal damage^43^. Accordingly we observed pathological swellings in the striatal terminals that morphologically resembled the axonal spheroids previously described in PD patients and in PD models (for review, see ref. 44). Axonal spheroids are considered a sign of disrupted axonal trafficking^45, 46^ a pathological event proposed to be relevant in the PD pathogenesis^47^. We did not observe co-accumulation of either neurofilaments or APP in the ASYN^+^ swellings. However, we did find co-accumulation of TH, VMAT2 and HSP70 mitochondrial protein in some of the larger round inclusions 12 weeks post-surgery, and occasionally of neurofilaments in axons of the MFB. Comparable accumulation of mitochondrial proteins has been found in round structures (*globules*) containing oxidized ASYN in ASYN transgenic mice^45^. Morphologically similar axonal pathology has been revealed by ASYN immunostaining in postmortem PD studies^48–50^. These axonal striatal swellings contained proteinase-K resistant ASYN^+^, an accepted marker of fibrillar insoluble ASYN, commonly used in human postmortem pathology^51–53^ and in animal models^8, 36, 53, 54^. Interestingly, proteinase-K resistant ASYN seems to selectively locate first in presynaptic terminals highlighting its possible role in synaptic dysfunction^55^. Furthermore, we showed the progressive accumulation in the axonal swellings of P-Ser129 ASYN, also considered pathological in α-synucleinopathy. Our analysis suggests that ASYN is first pathologically accumulated and later phosphorylated, or that the aggregates of ASYN later recruit P-Ser129-ASYN as previously suggested^18^.

With axonal damage as the primary event, the first signs of degeneration would be synaptic dysfunction, which can occur in surviving neurons with the behavioral consequences observed in the present study. Dopaminergic cell death is preceded by early loss of proteins involved in synaptic transmission and axonal transport in rAAV A53T mutant ASYN injected rats^12^. Lundblad *et al*. reported dysfunctional dopamine release that exceeded the loss of dopaminergic cell bodies in rats with ASYN overexpression via rAAV^10^. Moreover, overexpression of wild-type ASYN reduced the number of synaptic vesicles and dopaminergic synapses, impaired dopamine release and induced motor defects^56^. Similarly, we have *in vivo* microdialysis data showing an ASYN-induced decrease in the basal release of dopamine in the ipsilateral striatum in the rAAV-ASYN model in the absence of cell loss (Andersen, Zareba-Paslawska, Mørk, Romero-Ramos & Sotty, in preparation). Therefore, the ASYN pathology will disrupt the release of dopamine in the striatum, promoting thalamic inhibition by basal ganglia output nuclei and diminishing activation of the motor cortex. Dopamine depletion leads to increased spine size and decreased activation of cortical intratelencephalic pyramidal neurons, which innervate both the ipsilateral and contralateral striatum^57, 58^. Further, it has been recently shown that partial 6-OHDA lesions alter direct pathway medium spine neuron activity early and progressively, while indirect pathway medium spine neurons seem less affected^59^. Decreased bilateral engagement of direct pathway medium spine neurons by intratelencephalic pyramidal neurons may in turn release the inhibition that these medium spine neurons exert on the SNc^60^. The bilateral disinhibition of SNc neurons will result in increased dopamine release in the striatum, which in our model was likely observed as augmented VMAT2 levels as previously described^61^. Indeed, unilateral 6-OHDA lesions have been shown to induce a compensatory bilateral increase in dopamine release^62^. Moreover, bilaterally increased VMAT2 PET binding, as seen here, has been previously shown in the 6-OHDA and the lactacystin unilateral PD models^63^. In the ASYN rats reported here, only contralateral intact SNc neurons were efficiently able to increase VMAT2, since the pathological accumulation of ASYN in the ipsilateral SNc neurons may inadequately support dopaminergic neurotransmission and therefore compensatory mechanisms may fail. Several lines of evidence support this claim. First, while native ASYN is required to maintain normal neurotransmission^64, 65^, abnormal ASYN oligomers have been reported to interfere with the normal function^66^. Second, dysfunctional storage of dopamine in synaptic vesicles appears to play a major role in ASYN induced degeneration^67^. For example, a recent study in isolated striatal vesicles from PD patients showed a VMAT2-dependent defect in dopamine uptake^68^. Taken together, mishandled or excessive ASYN in the SNc will impair dopamine homeostasis and neurotransmission, leading to circuit-mediated bilateral changes and compensation in basal ganglia function. The restriction of ASYN to the SNc of one hemisphere will further interfere with dopamine-mediated compensations, leading to asymmetries in the system.

In conclusion, we demonstrated degeneration of nigrostriatal projections and asymmetric VMAT2 levels in the ASYN model of PD using non-invasive *in vivo* [^11^C]DTBZ PET. The evidence that synaptic disruption and initiation of motor deficits occurred in the absence of nigral cell loss indicates that early disruption of dopamine homeostasis and release is sufficient to affect basal ganglia and initiate motor symptoms related to the presence of pathological ASYN aggregation in axons. The evidence also demonstrates the advantages of this rodent model together with [^11^C]DTBZ PET for longitudinal testing of therapeutics that modulate dopamine homeostasis.

## Materials and Methods

### Animals

Female Sprague Dawley rats (n = 43) weighing 225–250 g at the time of surgery were housed two per cage under a 12 h light/12 h dark cycle at an average temperature of 21 °C, with *ad libitum* access to food and water. All procedures involving animals were conducted under humane conditions and were approved by the Danish Animal Experiments Inspectorate and in compliance with Danish laws and regulations for the Humane Care and Use of Animals in Research.

### Stereotaxic surgery of viral vectors

We expressed human wild-type ASYN, or GFP, in rat brain using rAAV2/6, with the transgene expressed under the synapsin-1 promoter and enhanced using a woodchuck hepatitis virus posttranscriptional regulatory element (WPRE). Final stock for both vectors was 1·10^13^ genome copies/ml (VectoBiolabs, USA). Plasmids were generously donated by Dr. Di Monte and Dr. Ulusoy (DZNE, Bonn, Germany). The animals received unilateral injections of the ASYN-rAAV2/6 (n = 23) or GFP-rAAV2/6 as control vector (n = 20). The animals were deeply anesthetized with medetomidine hydrochloride and fentanyl i.p. and then placed in a stereotaxic frame (Stoelting, Wood Dale, IL, USA). Two µl of rAAV (dilution 1·10^13^ genome copies/ml) were injected into the right SN (coordinates, 5.2 mm posterior, 2.0 mm lateral to bregma and 7.2 mm ventral relative to dura; nose −3.3), at a rate of 0.2 µl/30 sec using a glass cannula kept at target position for 5 additional min and then slowly retracted. For histological examinations, animals were killed at 4 and 12 weeks post-injection and a subset of animals were killed at 12 weeks for autoradiography.

### *In vivo* [^11^C]DTBZ positron emission tomography (PET)

DTBZ is a selective and robust ligand of the VMAT2^15, 25^. Animal brains were imaged with tracer [^11^C]DTBZ at 12 weeks post injection of rAAV-ASYN (n = 8) or rAAV-GFP (n = 5). Tracer [^11^C]DTBZ was synthetized as described previously^69^. Anesthesia was induced in a chamber filled with 2% isoflurane and maintained during dynamic acquisition through a mask for delivery of isoflurane (1.5–2%). Temperature, heart rate and respiration frequency were monitored throughout the study. Animals were placed prone in the microPET aperture (nanoScan® PET/MRI system, Mediso Ltd, Hungary, Budapest) (MRI image resolution 100 µm). We immobilized heads in a holder to obtain reproducible and comparable positioning of the brains. The average specific activity was 52.2 GBq/ml and the average injected mass was 2.47 microgram/ml, with no significant differences between the ASYN and GFP animals. The dynamic emission recordings began at the moment of injection of tracer [^11^C]DTBZ. The total acquisition lasted 90 min, with multiple frames increasing in duration from 15 sec. to 10 min. We based attenuation correction on individual MRI images by the material map segmentation method^70^. Dynamic data were reconstructed by filter back projection after attenuation and scatter correction. Dynamic emission recordings were summed and manually co-registered to the MRI atlas of rat brain with 6 degrees of freedom using the software Register (Montreal Neurological Institute, Montreal, Canada). Dynamic recordings were resampled to the common MRI space, the volume of interest (VOI) templates were used to extract time-activity curves for the brain regions, including cerebellum and left and right striatum. [^11^C]DTBZ distribution volume ratio (DVR) was estimated at times of 30–90 min using Logan plot with cerebellum serving as reference, assumed to have no displaceable component^71^. We calculated tracer [^11^C]DTBZ binding potentials (*BP*_ND_) as DVR-1, where DVR is the distribution volume ratio of specific to non-displaceable binding at steady-state^72^. *BP*_ND_ parametric maps were processed by voxel-wise normalization to cerebellum activity using the Logan plot^71^. To illustrate the anatomical location of the coronal, horizontal and sagittal slices of the parametric maps, we processed surface models based on the MRI atlas using the open-source 3D Slicer software (4.3.1 r225999).

### Autoradiography *in vitro* with [^3^H]DTBZ

We determined the striatal VMAT2 expression by autoradiography using the same ligand labeled with ^3^H (Perkin Elmer). Animals (n = 8 per group GFP & ASYN), were killed 12 weeks post rAAV surgery by decapitation and brains were quickly removed, frozen by immersion in isopentane at −40 °C and stored at −80 °C. Brains were sliced in 20 µm coronal sections on a cryostat (Vibratome ULTRApro 5000) at −15 °C, mounted on poly-L-lysine microscope slides (Menzel-Gläzer, Braunschweig, Germany) and stored at −80 °C until further processing. Eight striatal sections at the coronal level −0.3 mm from bregma were selected for [^3^H]DTBZ autoradiography and additionally four adjacent sections were selected for non-specific binding quantification. The slides were thawed at 25 °C for 30 minutes and pre-incubated in 40 mM Tris-HCl buffer at pH 8.2. We determined total binding by incubation with [^3^H]DTBZ at a final concentration of 7 nM in the same buffer for 90 min. The specific activity of the tracer was 20 Ci/mmol. Non-specific binding was measured by incubation in the same solution with the additional presence of 1 μM unlabeled DTBZ. After incubation, slides were rinsed by immersion in cold buffer (3 × 3 minutes) followed by a dip in distilled water. Slides were then dried under cold airflow and placed in a vacuum desiccator for 24 hours. The dried tissue slides were placed on [^3^H]-sensitive radioluminographic imaging plates (Fuji imaging plates, BAS, Fuji-Film) for 7 days with a ^3^H microscale (American Radiolabeled Chemicals, St. Louis). After exposure, the imaging plates were scanned (BAS-5000, Fuji Photo Film, Tokyo), and autoradiograms were analyzed using the ImageGauge 4.03 software (FujiFilm). Radioactivity concentrations in each side of striatum were obtained from autoradiograms extracted from regions of interest (ROI) drawn according to the stereotaxic atlas of the rat brain^73^. Non-specific binding was subtracted from total binding to obtain the specific binding. The specific relative to non-specific uptake ratio (SUR) was calculated in order to compare the autoradiography data with *in vivo* PET quantification.

### Analysis of behavior

We tested asymmetry in spontaneous forelimb use by the cylinder test, described previously^74^. We placed the animal in a transparent cylinder and video recorded the progress until it reached 20 vertical touches. Two mirrors were placed behind the cylinder to enable the recording of all forelimb touches. Data analysis was performed using the software VCL Media Player for replay in slow motion. Data are presented as percentage of the contralateral forelimb use compared to total number of touches. Forelimb akinesia was assessed with the stepping test^75^. The experimenter held the rat with both hind limbs plus one forelimb gently restrained in a fixed position, while allowing the unrestrained forelimb to touch the table. The animal was moved sideways along the table surface over a distance of 90 cm at an average speed of 18 cm/sec. The number of adjusting steps was counted both in forehand (i.e., the animal is moved left when the right paw touches the table and *vice versa*) and backhand direction (i.e., the animal is moved right when the right paw is touches the table and *vice versa*). The backhand stepping scores were used as a control for the animal’s performance, and the forehand steps were used to evaluate motor impairment^75, 76^. The test was performed twice a day for three consecutive days, and the data are presented as the average score from the last testing day. All analyses were done by an experimenter blind to the animal group identity.

### Histological analysis

Animals were overdosed with pentobarbital i.p. and perfused with saline through the ascending aorta, followed by 4% ice-cold paraformaldehyde. Brains were removed and post-fixed in the same solution for 2 hours, then transferred to 25% sucrose solution for cryoprotection, and subsequently sectioned on a freezing microtome in the coronal plane at a thickness of 40 µm. Immunohistochemical staining was performed on free-floating sections with the following primary antibodies: antibody raised against tyrosine hydroxylase (TH) (mouse, 1:3000, Chemicon, Temecula, CA), human ASYN raised against epitope 118–123 (rabbit, 1:4000,Abcam, Cambridge, UK), anti-GFP (rabbit, 1:200, Abcam, Cambridge, UK), and anti-VMAT2 (Goat, 1:2000, Millipore/Chemicon, Temecula, CA), DAT (rat IgG, 1:500, required heat antigen retrieval, Millipore/Chemicon, Temecula, CA), Ser129-phosphorylated human ASYN (1:1000; P-α-syn 1175; gift from Dr. Hasegawa^77^). Sections were rinsed in potassium-phosphate buffer (KPBS) between every incubation period and incubated in 0.25% Triton X-100 KPBS. Endogenous peroxidase activity was quenched for 10 minutes in a mixture of 3% H_2_O_2_ and 10% methanol in KPBS. The sections were pre-incubated 1 hour at room temperature with 5% normal relevant serum followed by overnight incubation with primary antibody in 2.5% normal serum. Thereafter sections were incubated 2 hours with appropriate biotinylated secondary antibody (1:200, Vector Laboratories, Burlingame, CA) in 1% normal serum, followed by avidin-biotin-peroxidase complex (ABC Elite, Vector Laboratories, Burlingame, CA). Sections were visualized using 3,3′-diaminobenzidine-tetrahydrochloride and 0.001–0.01% H2O2, mounted on glass slides coated with chrome-alum.

Immunofluorescence staining was performed on free-floating sections with a combination of two-three primary antibodies from the following list: antibody raised against human ASYN epitope 118–123 (rabbit, 1:4000, Abcam, Cambridge, UK), human ASYN clone 4B12 (mouse IgG, 1:500, Covance), TH (mouse IgG, 1:3000 Chemicon, Temeculxa, CA), VMAT2 (goat IgG, 1:5000, Everest Biotech), neurofilament 200 (rabbit IgG, 1:500, Sigma), amyloid precursor protein (APP) (rabbit IgG, 1:1000, Abcam), heat shock protein 70 (HSP70) (mouse IgG, 1:250, Abcam). Sections were rinsed in KPBS and incubated in 0.25% Triton X-100 KPBS. They were preincubated for one hour with 5% of relevant serum, followed by overnight incubation with primary antibody in 2.5% normal serum. They were then rinsed and incubated for 2 hours with appropriate Alexa Fluor conjugated secondary antibodies (568 rabbit IgG, 1:200; 488 mouse IgG, 1:400; 660 goat IgG, 1:200; 647 rabbit IgG, 1:500, 568 mouse IgG, 1:400, Invitrogen) in 1% normal serum. Sections were counterstained with DAPI (Sigma) and mounted on glass slides coated with chrome-alum and coverslipped with fluorescence mounting media and kept in the dark at 4 °C. The confocal-photos were taken using a Zeiss Lsm 710 microscope and the ZEN interface software.

### Proteinase K treatment

Proteinase-K resistance confirms the presence of aggregated ASYN amyloid. Sections were first rinsed in KPBS and endogenous peroxidase activity was quenched as described above, afterwards the sections were rinsed and mounted on glass slides coated with chrome-alum. The mounted sections were heated in an oven at 70 °C, thereafter incubated with proteinase-K (5 µg/ml, Thermo Scientific) in KPBS for 2 hours. As a control one slide with representative mounted section from each animal was incubated in KPBS without proteinase-K. After the proteinase-K treatment immuno-histochemical staining was performed as described above using a primary antibody raised against human and rat ASYN (sheep IgG, 1:800, Abcam) and a biotinylated secondary antibody (sheep IgG, 1:200) and developed using diaminobenzidine.

### Analysis of α-synuclein containing inclusions

ASYN-positive aggregates were quantified from coronal sections at +1.20 mm from bregma, immunostained for human ASYN. Two photos were taken per section of medial and dorsolateral areas of ipsilateral striatum. All quantifications of ASYN aggregates were performed in the software ImageJ 1.47 v and with a few modifications from the method previously described^53^. We measured the optical density of aggregates in individual animals, and applied the mean grey value as a threshold to segment aggregates. Thereafter, the number and size (µm^2^) of aggregates were obtained (circularity 0.3–1). Since the ASYN aggregates strictly do not appear in myelinated areas, and these areas can vary between animals, we also segmented individual images to measure the area covered only by ASYN containing fibers for normalization. Aggregates containing P-Ser129 ASYN were quantified from adjacent coronal sections immunostained for human anti-Ser129 ASYN. Several photos at 20x were taken and a super-image was created covering the full striatum. Thereafter, the same procedure as above was applied.

### Stereological and optical densitometry quantification

We performed unbiased stereological quantification of the number of TH^+^ cell bodies in SN, based on the optical fractionator principle with an accepted error coefficient of <0.10, as described previously^7^. Briefly, stereological counting was performed in sections that covered the full extent of SN from the rostral tip of the pars compacta to the caudal pars reticulata (about 8–9 sections per series) using the software NEWcast, Visiofarm (vs. 2.14.11.00). A low power objective lens (1.25x, SPlan) was used to outline the borders of the area of interest in SN, as described in detail previously^13^. The actual counting was performed with an objective at a magnification of 63x (NA 0.75).

TH, VMAT2 or DAT-stained striatal brain sections were scanned with the Epson Perfection 3200 Photo at a resolution of 2600 DPI. Densitometry analysis of striatal fibers was performed by measuring optical density at six coronal levels for TH^+^ fiber density (+1.6, +1.0, +0.2, +0.3, −0.9 and −1.4 mm from bregma), and three levels for VMAT2 ^+^ fibers (+1.0, −0.3 and −1.4 mm) and DAT^+^ fibers (+1.6, +0.2 and −0.3 from bregma) using ImageJ software (version 1.6.0, NIH, USA). The absolute values were then normalized for non-specific background staining by subtracting values obtained from white matter in corpus callosum. The data are presented as a percentage of the optical grey value of the intact contralateral striatum.

### Statistics

Statistical comparison of data used Graphpad Prism v. 7.00 for two-way factorial ANOVA with factors interaction and Tukey or Sidak post-hoc analysis and t-test when relevant. In all analyses, P < 0.05 indicated significance. See respective figure legends for details.

### Data availability statement

The authors agree to make all materials, data and associated protocols promptly available to readers without undue qualifications in material transfer agreements.

## Electronic supplementary material

Romero-Ramos Supplementary
