# Supplementary material for: Early synaptic dysfunction induced by α-synuclein in a rat model of Parkinson’s disease
Source: Sci Rep. 2017 Jul 25;7:6363. doi: 10.1038/s41598-017-06724-9 (PMC5526979; doi:10.1038/s41598-017-06724-9)
Supplement: Supplementary file 1 — Romero-Ramos Supplementary [file 41598_2017_6724_MOESM1_ESM.pdf]

Early synaptic dysfunction induced by α-synuclein in a rat model of Parkinson's Disease

Jenny-Ann Phan[1,2], Kathrine Stokholm[1,2], Justyna Zareba-Paslawska[1], Steen Jakobsen[2], Kim Vang[2], Albert Gjedde[3-8], Anne M. Landau*[2,9] and Marina Romero-Ramos*[1]

SUPPLEMENTARY MATERIAL

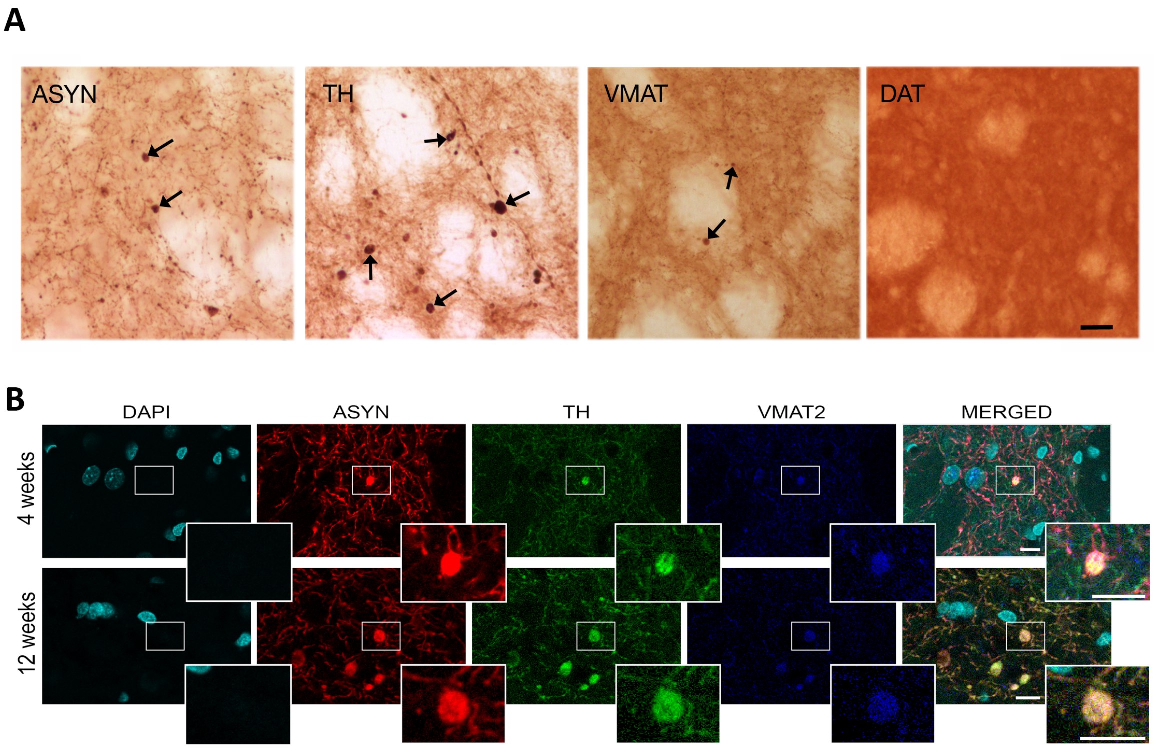

Supplementary Figure 1. Protein deposition in striatal fibers

High magnification photos from the ipsilateral striatum of one specific representative ASYN animal processed for histology 12 weeks post-surgery and immunostained for human ASYN, TH, VMAT2 or DAT (**A**). Both ASYN and TH immunostaining revealed pathological axonal swellings in the ipsilateral striatum of the rat (**A**; arrows). In comparison, in adjacent sections from the same animal immunostained for VMAT2, the formations were less numerous and only rare VMAT+ round swellings were found (arrows). However, in DAT immunostained sections, no pathological formations were obvious through the striatum of the same animal. Figure **B** shows representative confocal images of the ipsilateral striatum from ASYN animals processed for immunofluorescence staining at 4 and 12 weeks post-surgery. The analysis confirmed that ASYN (red) was expressed in dopaminergic TH^{+}(green) striatal fibers, and that the $ASYN^{+}$ pathological big accumulation contained both TH (green) and VMAT2 (blue) proteins. DAPI (Cyan) was used for counterstaining. Scale bar in the right top image = 25 μm applies to all in A, and in the most right confocal panels equals 10 μm.

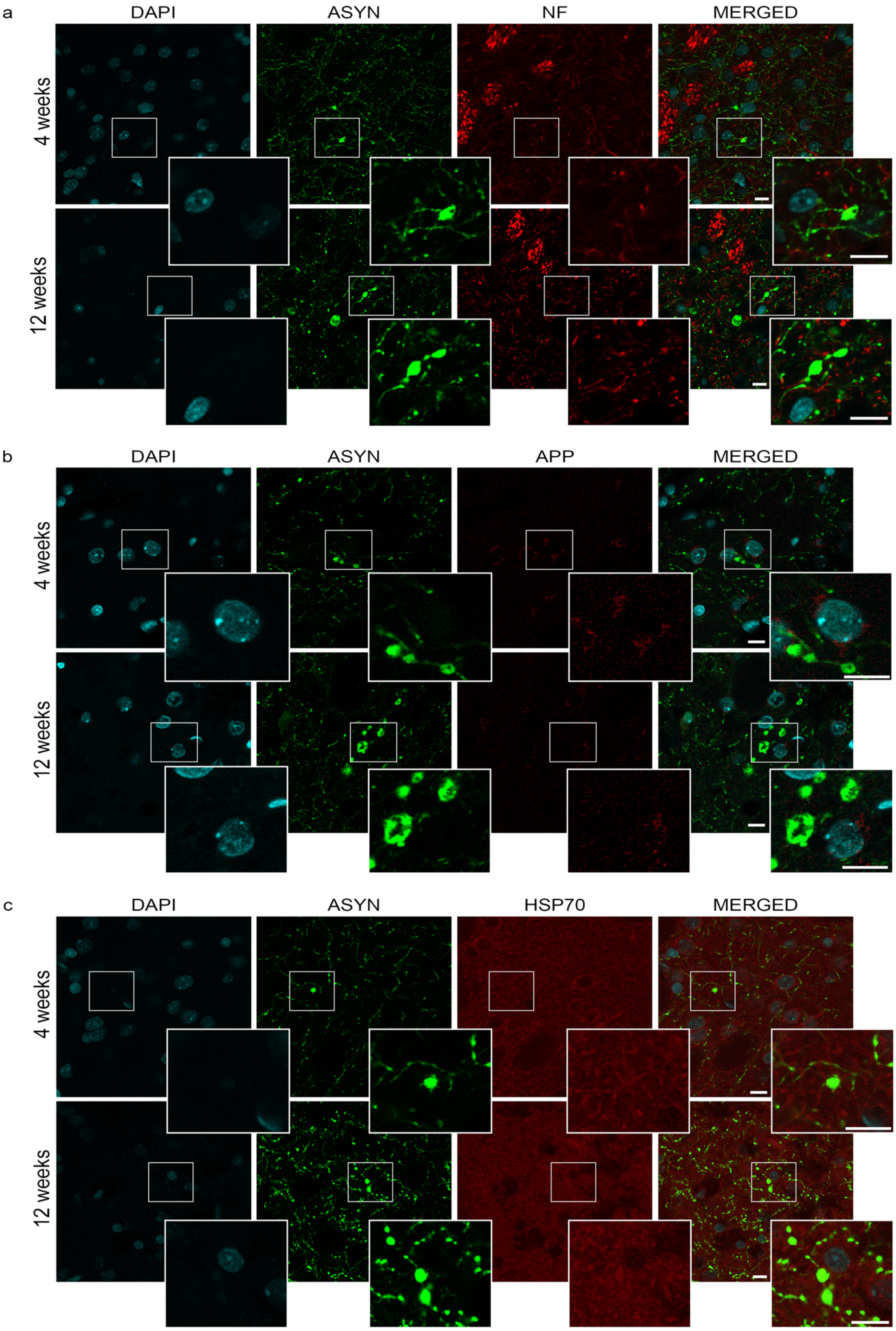

Supplementary Figure 2. Immunofluorescence - Striatum at 4 and 12 weeks post-surgery
Representative confocal images of the ipsilateral striatum from ASYN animals processed for immunofluorescence staining at 4 and 12 weeks post-surgery. (A) The analysis confirmed that pathological ASYN inclusions (green) did not contain overt accumulation of neurofilament (red) (B) nor APP (red) at any of the time points. (B) We found however that big ASYN+ swellings (green) contained the mitochondrial protein HSP70. DAPI (Cyan) was used for counterstaining in all cases. Scale bar= 10 µm applies to all.

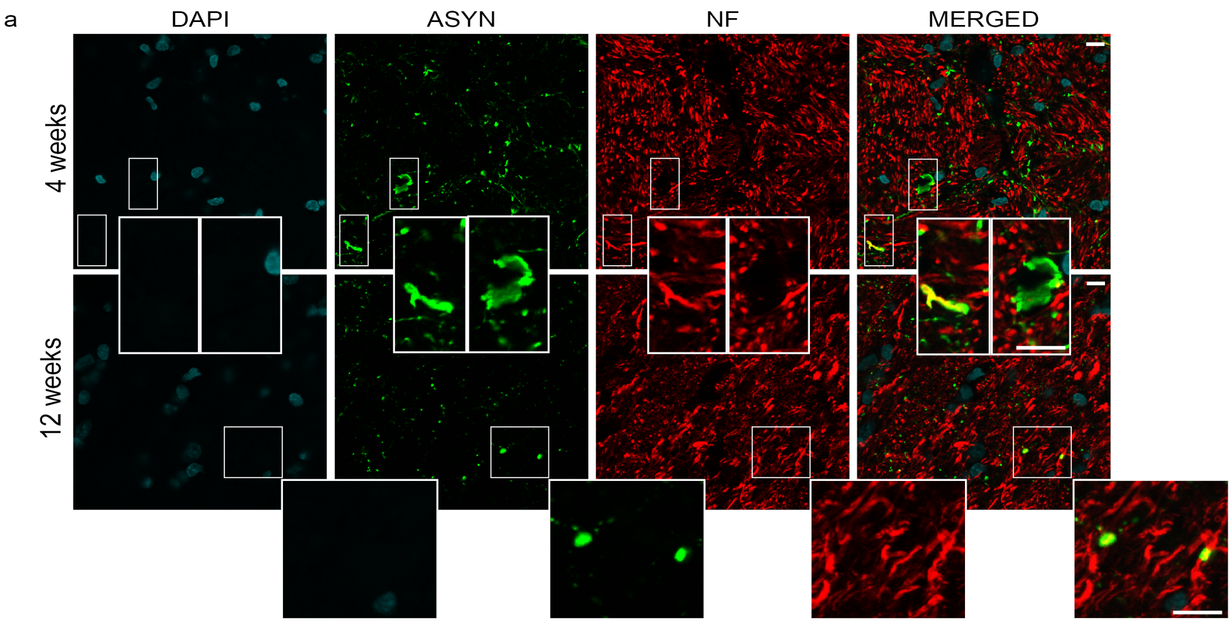

Supplementary Figure 3. Immunofluorescence - Medial forebrain bundle
Confocal photos of the ipsilateral medial forebrain bundle (MFB) from ASYN animals processed for immunofluorescence staining at 4 and 12 weeks post-surgery. The analysis confirmed that few pathological ASYN inclusions (green) in MFB also contained neurofilament (red) both at 4 and 12 weeks post-surgery. DAPI (Cyan) was used for counterstaining in all cases. Scale bar= 10 µm applies to all.
